# HDAC7 promotes ovarian cancer malignancy via AKT/mTOR signalling pathway

**DOI:** 10.1111/jcmm.70120

**Published:** 2024-10-21

**Authors:** Qi Feng, Sheng Hao, Xiongxiu Liu, Zhong Yan, Kai Sheng, Yanping Li, Peng Zhang, Xiugui Sheng

**Affiliations:** ^1^ School of Medicine Jinan University Guangzhou China; ^2^ International Cancer Center, Guangdong Key Laboratory of Genome Instability and Human Disease Prevention, Marshall Laboratory of Biomedical Engineering, Department of Biochemistry and Molecular Biology Shenzhen University Medical School Shenzhen China; ^3^ Department of Gastroenterology The First Affiliated Hospital, Jinan University Guangzhou Guangdong China; ^4^ Department of Gynecologic Oncology Linyi Cancer Hospital Linyi China; ^5^ Shenzhen Maternal and Child Healthcare Hospital Shenzhen China; ^6^ Institute of Precision Medicine Jining Medical University Jining Shandong China; ^7^ Shenzhen Institute of Advanced Technology, Chinese Academy of Sciences Shenzhen Guangdong China; ^8^ National Cancer Center/National Clinical Research Center for Cancer/Cancer Hospital & Shenzhen Hospital Chinese Academy of Medical Sciences and Peking Union Medical College Shenzhen China

**Keywords:** AKT, HDAC7, histone deacetylase, mTOR, ovarian cancer

## Abstract

Ovarian cancer is of the most lethal malignancy and causes serious threat to women health worldwide. A deep understanding of molecular mechanisms underlying ovarian cancer progression is critical for the development of promising therapeutic strategies. In this study, we aimed to employ immunohistochemistry to determine the protein level of HDAC7 in patient tissues, our data showed HDAC7 levels are upregulated in tumour tissues. In addition, we also performed Kaplan–Meier survival analysis to investigate the association between HDAC7 expression and clinical prognosis, and found that HDAC7 expression was associated with poor prognosis in ovarian cancer patients. Inhibition of HDAC7 cells resulted in lower cell proliferation, invasion and colony formation. Furthermore, we also found that HDAC7 inhibition suppressed PI3K/AKT/mTOR pathway. In contrast, exogenous HDAC7 expression activated the PI3K/AKT/mTOR pathway in HDAC7 knockout cells and rescued the cell proliferation, invasion and colony formation. However, inhibition of p‐AKT induced lower cell proliferation, metastasis and colony formation abilities. In murine model, HDAC7 KO significantly decreased the tumour burden. These data indicate that HDAC7 is involved in regulation of PI3K/AKT/mTOR pathway and targeting of HDAC7 could be potential therapeutic strategy in the treatment of ovarian cancer.

## 1 INTRODUCTION

Ovarian cancer (OC) remains the most common gynaecological tumour with nearly 19,710 estimated new cases and 13,270 deaths yearly in United States.^1^, ^2^ Although past few decades witnessed great progress in prevention and treatment of the ovarian cancer, the survival rate of ovarian cancer patients is still poor because of frequent metastasis and rapid cell proliferation.^3^, ^4^, ^5^ To data, the standard care for ovarian cancer is cytoreductive surgery followed by chemotherapy.^6^, ^7^ There has been evidence that chemotherapy is promising at early tumour stage; however, most patients at advanced stages demonstrated chemotherapy resistance and recurrence.^8^, ^9^, ^10^ Therefore, the molecular mechanism underlying ovarian cancer progression and recurrence is urgently needed to be further elucidated for developing new strategies in the treatment of ovarian cancer.

Recently, idea of targeting histone deacetylases (HDACs), which consist of 18 proteins and have been classified into four subcategories based on their sequence homology to the yeast,^11^, ^12^ Class I (HDAC1/2/3/8), class IIa (HDAC4/5/7/9), Class IIb (HDAC6/10), class III (sirt1‐7) and class IV (HDAC11),^13^, ^14^, ^15^ has emerged as a promising treatment strategy for solid tumours.^16^, ^17^, ^18^ HDACs regulate various aspects of cell biology, including proliferation, angiogenesis and migration.^19^, ^20^, ^21^ Aberrant HDACs expressions, especially HDAC7 is implicated in the onset and progression of many cancers.^15^, ^22^, ^23^, ^24^ HDAC7 belongs to class IIa HDACs and contributes in the progression of breast cancer, nasopharyngeal cancer and lung cancer.^12^, ^15^, ^25^ HDAC7 promotes cell proliferation, migration and stemness in the pathological and physiological conditions.^18^, ^26^ High HDAC7 expression is associated with metastasis and poor prognosis.^27^, ^28^ What' more, HDAC7 is also one of targets among various HDAC inhibitors, for example Trapoxin (TPX)‐A and ‐B, Containing peptides (CHAPs), Phenacetyl hydroxamates and 2‐phenylbenzoyl hydroxamates.^29^, ^30^, ^31^ However, the role and molecular mechanism of HDAC7 underlying the oncogenic function in ovarian cancer have been poorly studied. To improve clinical implementation and develop potential drugs, the exact function and detailed mechanism of HDAC7 in ovarian cancer progression are urgently needed to be further explored.

In the present study, we have investigated the key role of HDAC7 in ovarian cancer cell proliferation, colony formation. We first unmasked that HDAC7 acts as an oncogene in ovarian cancer by promoting AKT/mTOR pathway. We found that HDAC7 expression is associated with poor prognosis of ovarian cancer patients and induces cell proliferation and invasion by activating AKT/mTOR pathway. Mechanistically, HDAC7 enhanced ovarian cancer progression by activating p‐AKT and p‐mTOR. HDAC7 knockout significantly suppressed cell proliferation, colony formation and invasion by decreasing the phosphorylation levels of AKT and mTOR. These findings show novel evidence to exploit HDAC7 as a potential therapeutic target in ovarian cancer.

## 2 MATERIALS AND METHODS

### 2.1 Tissue specimens

Ovarian tissue microarray slides, containing tumour and normal tissues (*n* = 5) were obtained from National Cancer Center/Cancer Hospital & Shenzhen Hospital. The study protocols and the use of archived cancer tissues were approved by The Ethics Committee of National Cancer Center/Cancer Hospital & Shenzhen Hospital.

### 2.2 Immunohistochemistry (IHC)

IHC was carried out according to manufacturer's instructions. Tissues antigen was retrieved following deparaffinized in xylene and rehydrated in alcohol, followed by heating in citrate buffer (Sangon Biotech, A601183, Shanghai, China). After that, slides were incubated in 3% hydrogen peroxide (ThermoFisher, 3587191, USA) for 15 min to inhibit the endogenous activity of peroxidase activity and then treated with 5% horse serum for 20 min at room temperature (Bioss Biotechnology Company, C‐0005, Beijing, China). Later, tissues were incubated in primary antibody HDAC7 (ab12174, Abcam, 1:100) at 4°C for overnight. After secondary antibody was incubated on the sections for 20 min at room temperature, horseradish peroxidase (NEOBIOSCIENCE, ZH0618, shenzhen, China) was incubated other 20 min. washing sections with PBS, and then the tissues were visualized by diaminobenzidine (NEOBIOSCIENCE, IHC DAB, shenzhen, China). Images were aquired by a microscope.

### 2.3 Cell culture and generation of HDAC7 knock out and mutant cell lines

Human ovarian cancer cells ES2 and OVCAR‐3 (Shanghai EK‐Bioscience Biotechnology Co., Ltd) were, respectively, cultured in McCoy's 5A medium (Basal Media, L630KJ, China) and RPMI 1640 medium (Gibco, 11875119, USA) supplemented with 10% fetal bovine serum (FBS) at 37°C in 5% CO_2_. The oligonucleotides utilized to the targeted sequences were synthesized by Tsingke (Guangzhou, China). The targeted sequences of human HDAC7 were as follows: HDAC7 sgRNA‐1: 5′‐CACCGGGGGCTCCGGAGCCAGTGTG‐3′; HDAC7 sgRNA‐1: 5′‐CACCGGGGACCAGATGCTCTGGAT G‐3′ and Lenti‐CRISPR V2 puro vectors (Addgene) as NC (negative control). HDAC7 mut (mutant restore) F: 5′‐CAGCAGAGCAAGGCCAGCAAGATACTGATAGTTGACTGGGACGTGCACCATGGC‐3′; HDAC7 mut (mutant restore) R: 5′‐GCCATGGTGCACGTCCCAGTCAACTATCAGTATCTTGCTGGCCTTGCTCTGCTG‐3′ and pCDH puro vectors (Addgene) as NC (negative control). Lentivirus was produced in 293FT cells by following manufacturer's instructions. Finally, HDAC7 protein level was determined in transfected ES2 and OVCAR‐3 cell lines by western blot analyses.

### 2.4 Reagents and antibodies

MK‐2206 2HCl was purchased from Selleck (Cat #::1032350‐13‐2, Shanghai, China) and dissolved in DMSO for invitro experiments. Antibodies against HDAC7 (Cat #: 10831), p‐mTOR (Cat #: 5536), mTOR (Cat #: 2983), p‐AKT (Cat #: 4060), AKT (Cat #: 4691) and β‐actin (Cat #: CST3700) were purchased from Cell signaling technology. Secondary antibodies (anti‐Mouse IgG, Cat #: 7076; anti‐Rabbit IgG Cat #: 7074) were also purchased from Cell signaling technology (USA).

### 2.5 Western blotting

Briefly, total protein was extracted from ES2 and OVCAR‐3 by using cell lysis buffer (1.5 M NaCl, 1 M HEPES [pH = 7.0], 1% NP40, 0.1 M Na_4_P_2_O_7_, 0.1 M NaF, 0.1 M Na_3_VO_4_, protease inhibitor). 15–20 μg protein was loaded and resolved by 10% electrophoresis, transferred to polyvinylidene fluoride membranes, (Millipore, USA) and incubated with primary antibodies against HDAC7 (1:1000), p‐mTOR (1:5000), mTOR (1:3000), p‐AKT (1:3000), AKT (1:1000) and β‐actin (1:10000). After wash, the secondary antibodies were incubated for 1.5 h and then signal were detected by Chemiluminescence detector (Tanon 5200CE).

### 2.6 Cell proliferation and colony formation assays

Cell proliferation was measured by the cell counting after 6 days of seeding 5 × 10^4^ cells in 6‐well plates. The colony formation was measured by crystal violet staining after 2 weeks of seeding 2 × 10^3^ cells in 12‐well plates.

### 2.7 Matrigel invasion assays

Invasion potential of the ovarian cancer cells ES2 and OVCAR‐3 was measured using Matrigel invasion assay. The polycarbonate transwell filter (Corning Inc., CLS3422) chamber was incubated at serum‐free medium for 2 h at RT. After that, seeded 5 × 10^4^ infected cells (ES2 and OVCAR‐3 cells infected with V2, HDAC7 sgRNA or pCDH‐HDAC7‐mut) into the upper chamber on a transwell filter equivalently and incubated in 1% serum medium for 24 h. Then, invasive cells on the lower surface of the filter were fixed by using 4% paraformaldehyde and stained with 0.3% crystal violet. Finally, images captured at 200× magnification with five random fields were assessed for every transwell filter.

### 2.8 Tumour xenograft study

Approximately, 1 × 10^6^ ES2 cells infected with lentiviral vectors were subcutaneously injected into the belly (left‐V_2_, right‐HDAC7 sgRNA) of 4‐week‐old NOD‐ Prkdc^scid^ IL2rg^tm1^/Bcgen mice. 30 days later, tumour volume and weight were measured.

### 2.9 RNA sequencing and analysis

Control and HDAC7 knockout in OVCAR‐3 cells were used for transcriptome RNA sequencing. The transcriptome sequencing and analysis were performed by Beijing Novogene Bioinformatics Technology Company (Beijing, China). Firstly, mRNA was purified from total RNA by using poly‐T oligo‐attached magnetic beads, and cDNA was synthesized using random hexamer primer and M‐MuLV Reverse Transcriptase. Then PCR was performed with Phusion High‐Fidelity DNA polymerase, and PCR products were purified (AMPure XP system), and library quality was assessed on the Agilent Bioanalyzer 2100 system. Lastly, the library preparations were sequenced on an Illumina Novaseq platform and 150 bp paired‐reads were generated. Reads were mapped to Homo sapiens (hg38/GRCh38) using Hisat2. The expression value of gene PFKM was quantified by using Feature counts v 1.5.0‐p3 software. Differential expression analysis was performed using the DESeq2 R package. The result *p*‐values were adjusted using the Benjamini and Hochberg's approach for controlling the false discovery rate. Genes with an adjusted *p*‐value <0.05 found by DESeq2 were assigned as differently expression. Gene Ontology (GO) and KEGG enrichment analysis of differently expressed genes was carried out by the clusterProfiler R package. Principal component analysis (PCA) of differently expressed genes was implemented by DESeq2 R package. The RNA‐seq data have been uploaded in the Sequence Read Archive (SRA) repository at NCBI under the accession number PRJNA1098488.

### 2.10 Real‐time quantitative polymerase chain reaction assay

TRIzol Reagent (Invitrogen, USA, 15596018) was used to isolate the total high‐quality RNA from HDAC7 knockout and V2 (negative control) cell line. Reverse transcription of total RNA to cDNA was performed by using PrimeScript RT (TAKARA, China, RR047A). After this, Real‐time quantitative polymerase chain reaction system was fellowed the protocol of SYBR Green qPCR Master Mix (Selleck, China, B21202) and performed by using the CFX96™ real‐time PCR detection system (Bio‐Rad, USA) in triplicate. β‐actin was used as reference gene control. The specific sequences of the RT‐qPCR primers were show in Table S1.

### 2.11 Statistical analysis

All statistical analyses were performed by using Student's *t*‐test. Each experiment was performed in triplicate. Data were presented as mean ± standard deviation (mean ± SD). *p*‐value <0.05 was defined as statistically significant difference.

## 3 RESULTS

### 3.1 HDAC7 is upregulated in ovarian cancer and high HDAC7 expression correlates with poor prognosis

**FIGURE 1:**
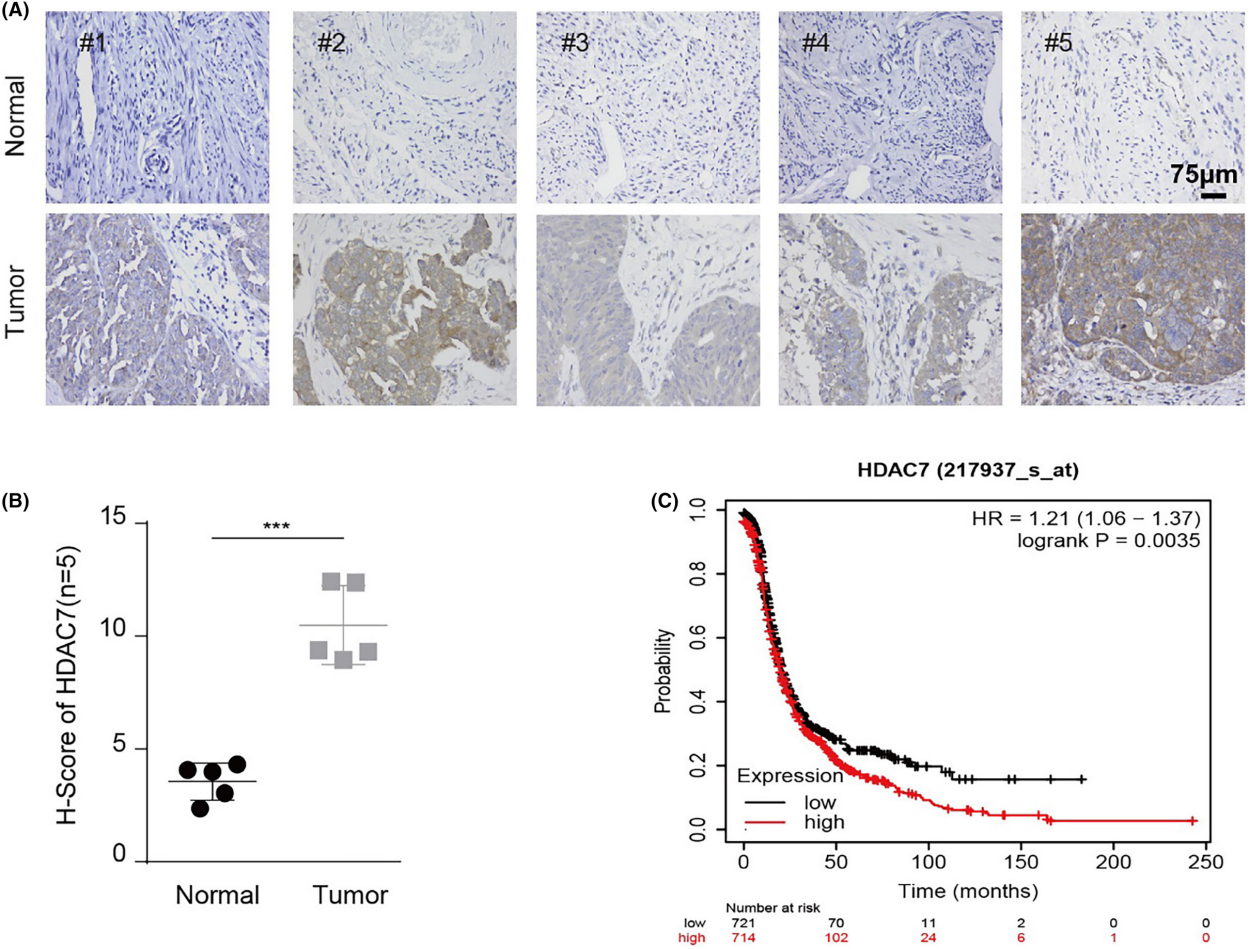
HDAC7 is upregulated in ovarian cancer and high HDAC7 expression correlates with poor prognosis. (A) Representative images of HDAC7 IHC staining and (B) H‐scores (mean ± SD) in sections of fallopian tube (*n* = 5) and ovarian tumours (*n* = 5), each group from the same patient; (C) Kaplan–Meier analysis the effect of HDAC7 on survival in ovarian cancer; The cell experiment was repeated 3 times. The data were expressed as mean ± standard deviation and analysed by the *t* test (*p < 0.05; **p < 0.01; ***p < 0.001).

To investigate the role of class IIa HDACs in ovarian cancer, we first analysed their mRNA expression levels and the relationship between their expression levels and prognosis by TCGA database, and then found that only HDAC7 expression was positively correlated with the tumour malignancy grade and that its high expression predicts poor prognosis (Figure S1). Furthermore, we determined HDAC7 expression in both the tumour tissues and normal tissues by Immunohistochemistry (IHC), and found that HDAC7 is upregulated in tumour compared with normal tissue (Figure 1A). Moreover, statistical analysis also suggested that HDAC7 protein expression in tumours was higher than that in normal tissues (Figure 1B). Next, we rechecked the association between HDAC7 expression and ovarian cancer patients' survival by using Kaplan–Meier plotter database, and found that HDAC7 expression is associated with poor survival in ovarian cancer patients (Figure 1C). These data indicated that HDAC7 expression is elevated in ovarian cancer tissue and negatively correlates with ovarian cancer patients' prognosis.

### 3.2 HDAC7 inhibition suppresses ovarian cancer cell proliferation, colony formation and invasion and depresses phosphorylation level of AKT/mTOR in vitro

**FIGURE 2:**
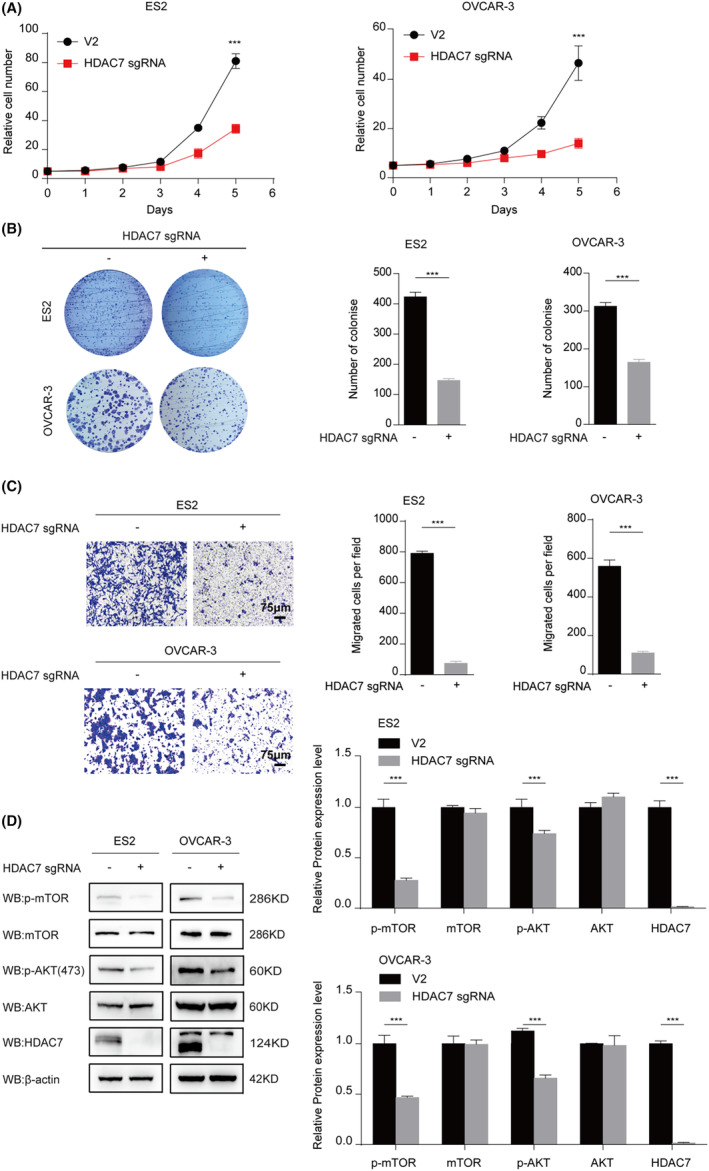
HDAC7 inhibition suppresses ovarian cancer cell proliferation, colony formation and invasion and depresses phosphorylation level of AKT/mTOR in vitro. (A) The cell proliferation of HDAC7 knockout cell lines were measured by cell counting; (B) Colony formation assay was used to measure the ability of cell generations; (C) Transwell migration assay was measured by inverted microscope (left) and counting analysis (right), 200 ×, scale bar = 75 μM in HDAC7 knockout groups and control groups. Cells with HDAC7 knockout show decreased invasion, compared to control; (D) The levels of AKT/mTOR signal pathway and HDAC7 were measured by Western blot; The cell experiment was repeated 3 times. The data were expressed as mean ± standard deviation and analysed by the *t* test (*p < 0.05; **p < 0.01; ***p < 0.001).

The observed upregulation of HDAC7 in tumour tissues and its association with poor prognosis in patients with ovarian cancer led us to postulate that HDAC7 may act as tumour promoter. In this line, we established stable HDAC7 knockout cell lines OVCAR‐3 and ES2 by lentivirus and performed cell counting and colony formation assays to determine the role of HDAC7 inhibition in regulating cell proliferation of OVCAR‐3 and ES2 cells in vitro. The results indicated that knockout of HDAC7 significantly inhibited the cell proliferation and colony formation abilities of OVCAR‐3 and ES2 (Figure 2A,B). Next, we performed transwell assay to explore the role of HDAC7 knockout in regulating invasion of ovarian cancer. The results suggested that depletion of HDAC7 in human ovarian cancer cells significantly inhibited invasion of OVCAR‐3 and ES2 cells (Figure 2C). After establishing the critical role of HDAC7 in regulating OVCAR‐3 and ES2 cells proliferation and invasion, we were interested in exploring underlying molecular mechanism. For this purpose, we determined the activation of pro‐survival AKT/mTOR pathway already reported to be engaged in ovarian cancer progression. Interesting, we found that HDAC7 inhibition blocked the AKT/mTOR pathway by decreasing the phosphorylation levels of p‐AKT(S473) and p‐mTOR (S2448) in OVCAR‐3 and ES2 cell lines (Figure 2D). To further explore the molecular mechanism about HDAC7 and AKT, a flag‐pulldown experiment was employed to detect the interaction between AKT and HDAC7. Unfortunately, HDAC7 did not bind AKT directly (Figure S2A). Therefore, we speculate HDAC7 may regulate the activation of AKT by regulating upstream signalling pathway of AKT. To address this hypothesis, we have performed RNA‐seq assay and then bioinformatics analysis was performed. The results demonstrated that the expression of many mRNA was significantly altered as shown in the heat map and Volcano plot (Figure S2B,C). What's more, principal component analysis (PCA) showed that there were clearly differences of mRNA expression between control and HDAC7 knockout group (Figure S2D). To further explore, how HDAC7 regulates AKT signalling pathway, we found all the significantly expressed genes related to AKT signalling in RNA‐seq data (Figure S2E), and we next performed qPCR assay to verified the differences of mRNA expression. Our results showed that mRNA levels of PTEN and PPP2R2B, suppressors of AKT/mTOR signalling, were upregulated and mRNA level of PIK3CB activators of AKT/mTOR signal pathway, was downregulated when HDAC7 was knockout (Figure S2F). Taken together, these novel findings suggested that inhibition of HDAC7 suppressed ovarian cancer cell proliferation, colony formation and invasion by reducing phosphorylation levels of AKT/mTOR in vitro.

### 3.3 HDAC7 promotes ovarian cancer malignant progression by regulating AKT/mTOR signal pathway

**FIGURE 3:**
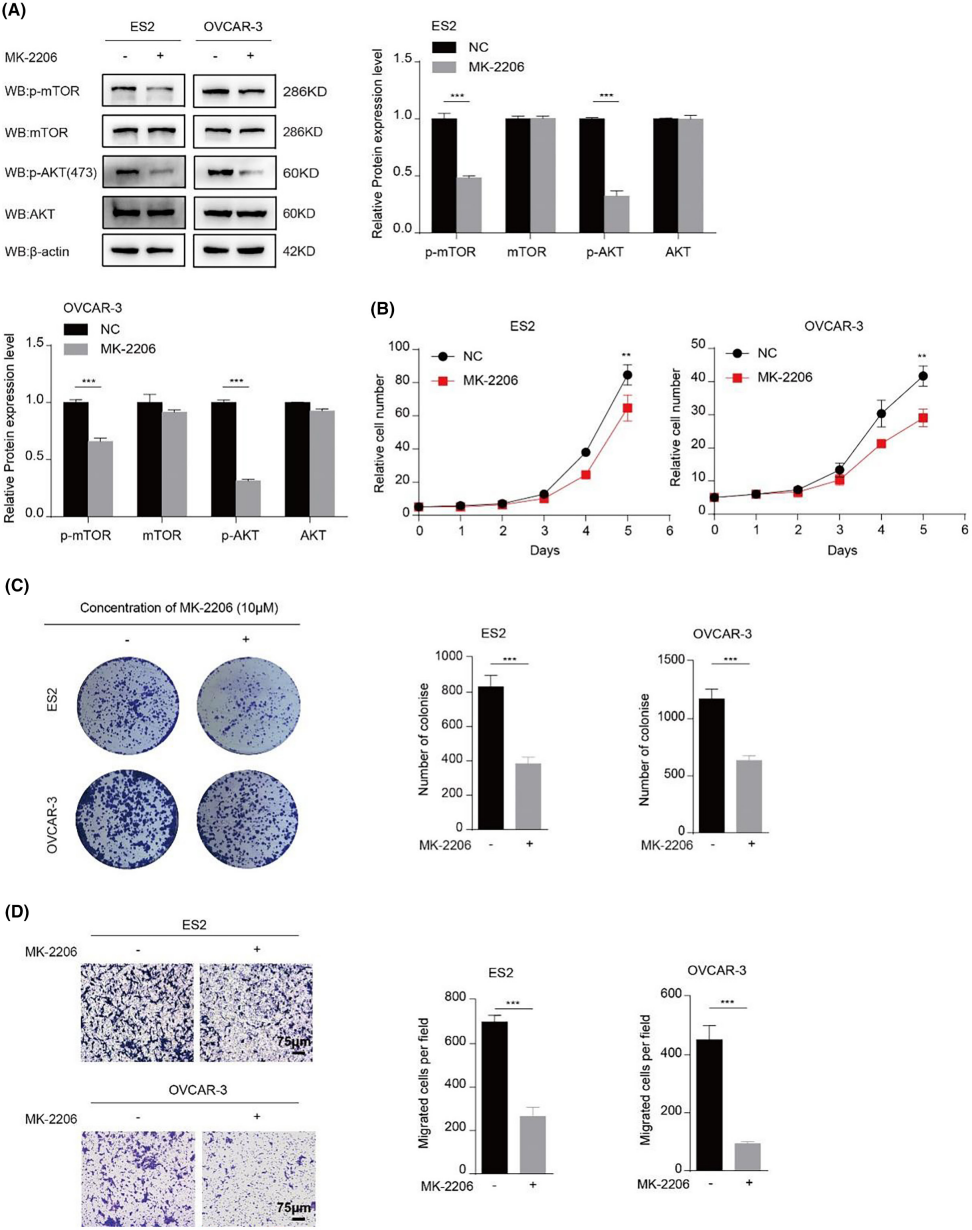
HDAC7 promotes ovarian cancer malignant progression by regulating AKT/mTOR signal pathway. (A) The levels of AKT/mTOR signal pathway were measured by Western blot, after treated with MK‐2206 2HCl for 24 h; (B–D) The cell proliferation (B), Colony formation assay (C) and Transwell migration assay (D) of ES2 and OVCAR‐3 cell lines treated with MK‐2206 2HCl; The cell experiment was repeated 3 times. The data were expressed as mean ± standard deviation and analysed by the *t* test (*p < 0.05; **p < 0.01; ***p < 0.001).

To further clarify that HDAC7 promotes ovarian cancer progression via regulating AKT/mTOR signalling, we treated OVCAR‐3 and ES2 cells with MK‐2206 2HCl, an AKT inhibitor and tested the expression pattern of AKT/mTOR‐related proteins such as p‐AKT and p‐mTOR. Results of Western blot analysis indicated that p‐AKT(S473) and p‐mTOR(S2448) levels were remarkably decreased in OVCAR‐3 and ES2 upon MK‐2206 2HCl treatment (Figure 3A). We also detected OVCAR‐3 and ES2 cell proliferation and invasion following MK‐2206 2HCl treatment. These results showed that AKT/mTOR pathway inhibition suppresses OVCAR‐3 and ES2 cell proliferation and invasion (Figure 3B–D). What's more, mutant activation of AKT was generated in a context of HDAC7 inhibition to fully demonstrate that HDAC7 promotes the malignant progression of ovarian cancer through the AKT/mTOR pathway. Strikingly, the results showed a rescue of the tumorigenic phenotype in vitro (Figure S3). Excitingly, the similar phenotypes have been observed in vivo (Figure S4). Overall, above data demonstrate that HDAC7 fuels ovarian cancer progression by regulating the AKT/mTOR pathway.

### 3.4 Ectopic expression of HDAC7‐mut eliminates the effects of HDAC7 knockout in ovarian cancer cells

**FIGURE 4:**
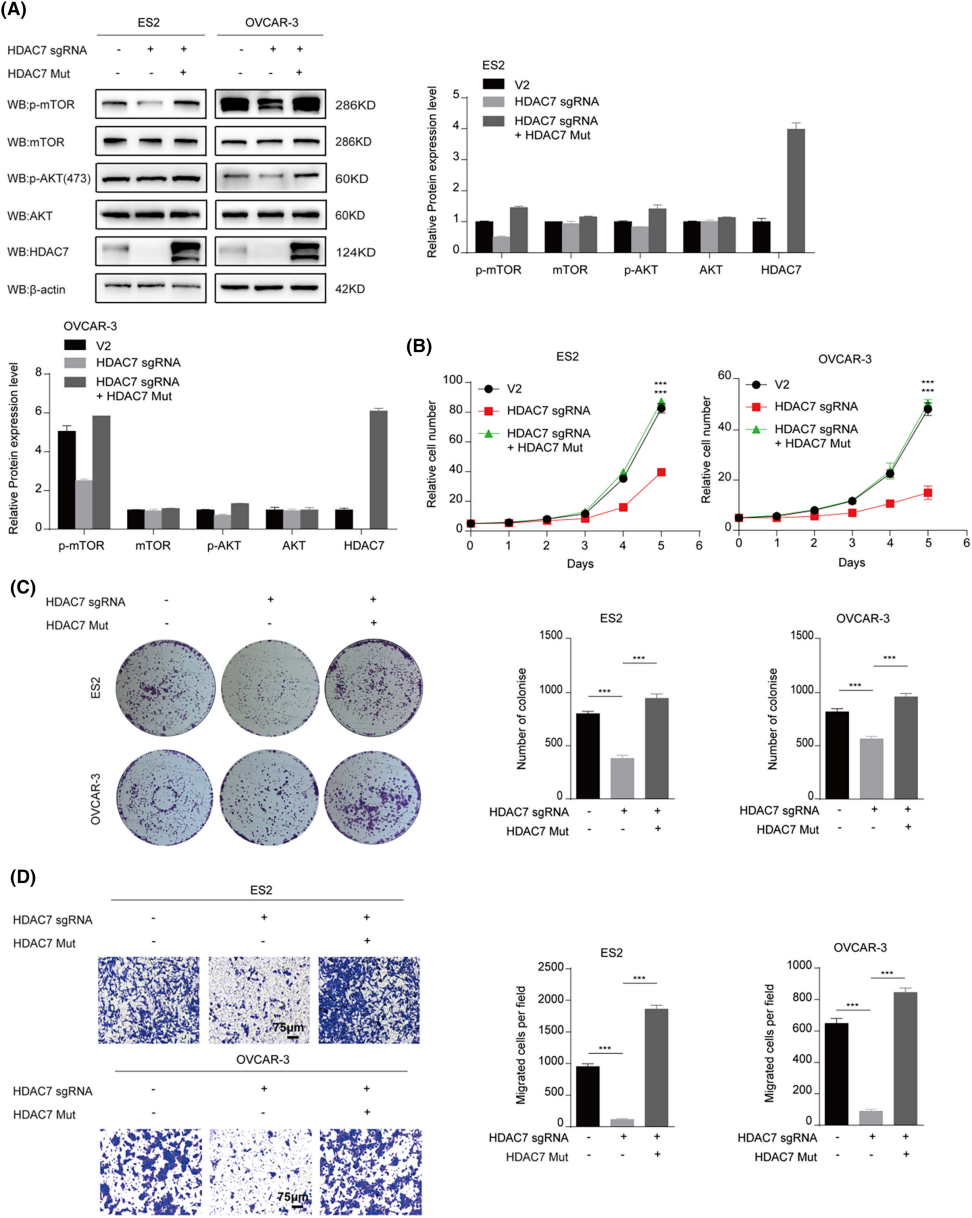
Ectopic expression of HDAC7‐mut eliminates the effects of HDAC7 knockout in ovarian cancer cells. (A) The levels of AKT/mTOR signal pathway and HDAC7 were measured by Western blot in HDAC7 knockout or HDAC7‐mut and control groups. Cells with HDAC7‐mut show rescue the level of p‐AKT(S473) and p‐mTOR(S2448), compared to HDAC7 knockout groups; (B–D) The cell proliferation (B), Colony formation assay (C) and Transwell migration assay (D) of ES2 and OVCAR‐3 cell lines which transfected HDAC7 knockout or HDAC7‐mut Lentiviral; The cell experiment was repeated 3 times. The data were expressed as mean ± standard deviation and analysed by the t test (*p < 0.05; **p < 0.01; ***p < 0.001).

**FIGURE 5:**
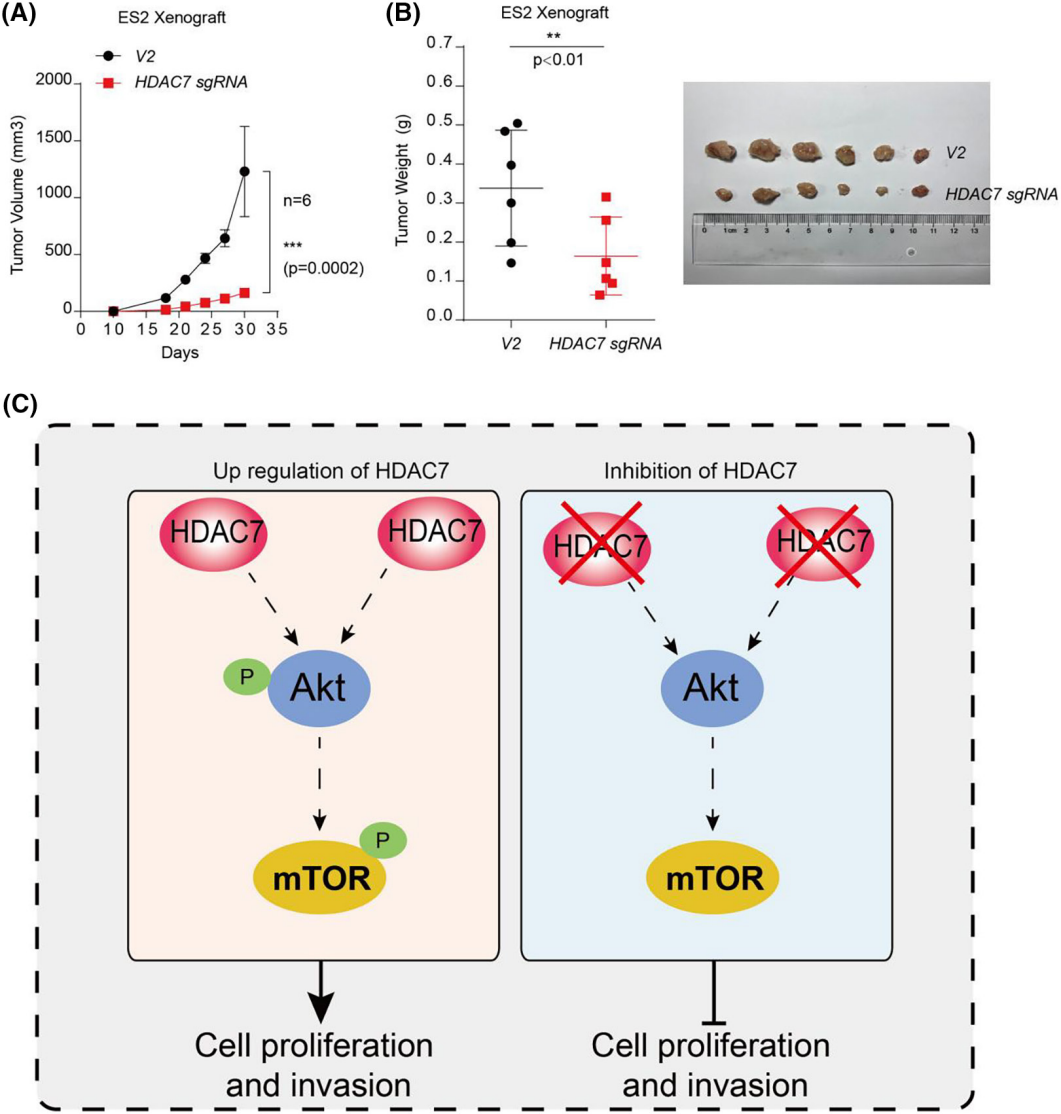
Inhibition of HDAC7 depresses cancer cell growth in vivo. (A) NOD‐ Prkdc^scid^ IL2rg^tm1^ /Bcgen mice were orthotopically injected with ES2 cells (1 × 10^6^ cells/mouse in 150 μL volume), which were infected with the indicated lentiviral vectors. Tumour volumes were measured by vernier calliper every 3 days until the end of the experiment; (B) The tumours were isolated and pictured (right) and tumour weight (left) was calculated in control group and HDAC7 KO group (*n* = 6 mice/group) for 30 days after injected; (C) A schematic model shows that HDAC7 facilitates ovarian cancer cell proliferation and invasion by activating PI3K/AKT/mTOR signalling; The data were expressed as mean ± standard deviation and analysed by the *t* test (*p < 0.05; **p < 0.01; ***p < 0.001).

To further test the relationship between HDAC7 and AKT/mTOR pathway, HDAC7 knockout cell lines were transfected with HDAC7‐mut, which is resistant to HDAC7 sgRNA, to rescue the expression of HDAC7 though Lentiviral technology. As shown (Figure 4A), ectopic expression of HDAC7 in HDAC7 KO cells rescued AKT/mTOR pathway activation in both cell lines by increasing phosphorylation levels of p‐AKT(S473) and p‐mTOR(S2448). What's more, higher cell proliferation and invasion abilities were observed upon overexpression of HDAC7‐mut in HDAC7 KO cell lines compared with HDAC7 KO groups (Figure 4B–D). Excitingly, these results suggested that Ectopic expression of HDAC7‐mut can rescue the effects of HDAC7 knockout in ovarian cancer cells in vitro. Finally, inspired by our previous data, we hypothesized that in vivo the similar story will take place. To test this hypothesis, a murine xenograft model was established. Following the xenograft of ovarian cancer cells, tumours were allowed to grow without any treatment for 4 weeks. After this, as demonstrated in (Figure 5E,F), the tumour growth, volume and weight were significantly decreased in HDAC7 KO groups compared with control groups, thereby suggesting inhibition of HDAC7 could depress cancer cell growth in vivo.

Collectively, these data provide strong evidences and new sights that HDAC7 controls ovarian cancer cell proliferation and invasion by regulating AKT/mTOR pathway. In the terms of mechanism, inhibition of HDAC7 induces remarkably decrease at the levels of p‐AKT and p‐mTOR, which is extremely important for tumour progression, resulting in arresting of cell proliferation and invasion. In addition, this study revealed that HDAC7 promotes ovarian cancer cell proliferation and invasion by augmenting the phosphorylation of AKT/mTOR and HDAC7 expression is positively correlated with poor prognosis (Figure 5G).

## 4 DISCUSSION

Ovarian cancer remains the most deadly gynaecological tumour and second leading cause of gynaecological tumour‐associated deaths among women worldwide owing to poor understanding of the underlying molecular mechanism of ovarian cancer progression.^7^ Therefore, investigation of molecular mechanism underlying ovarian cancer progression is urgently needed for the development of promising clinical management approaches. Here, we found that HDAC7 is highly expressed in ovarian cancer and positively correlated with invasion and poor prognosis. HDAC7 inhibition significantly suppressed growth and invasion of the ovarian cancer cells in vitro and depressed tumour growth in vivo, suggesting that HDAC7 has a significant contribution in ovarian cancer progression and may function as an oncogenic driver of ovarian cancer.

Previous studies reported the oncogenic roles of HDAC7 in several cancers types including lung cancer and leukaemia and it plays a vital role in regulating activity of FGF‐18 and c‐Myc.^12^, ^32^ However, no study has been conducted yet to investigate whether HDAC7 regulate the classic AKT/mTOR pathway, which plays an indispensable role in the progression of tumour. Here, we reasoned to explored the function of HDAC7 in ovarian cancer progression and found that inhibition of HDAC7 obviously cut down p‐AKT and p‐mTOR phosphorylation levels and induced a low rate of cell proliferation and invasion. What's more, mut‐activated AKT in ovarian cancer, can rescued cell proliferation and invasion which cause by HDAC7 inhibition. These findings demonstrated that HDAC7 controls the progression of ovarian cancer by regulating positively regulates the AKT/mTOR pathway.

In conclusion, we elucidated the underlying molecular mechanism of HDAC7 in the progression of ovarian cancer growth via enhancing the levels of p‐AKT and p‐mTOR. Although present study has shown novel findings; however, some limitations can't be neglected. The main limitation of present study is that how HDAC7 regulates the phosphorylation of AKT. Therefore, it needs to be investigated in future study.

## AUTHOR CONTRIBUTIONS

**Qi Feng:** Conceptualization (lead); data curation (equal); formal analysis (equal); funding acquisition (equal); investigation (equal); methodology (equal); project administration (equal); writing – original draft (equal). **Sheng Hao:** Conceptualization (equal); data curation (equal); formal analysis (equal); investigation (equal); methodology (equal); writing – original draft (equal). **Xiongxiu Liu:** Formal analysis (supporting); methodology (supporting). **Zhong Yan:** Formal analysis (supporting); methodology (supporting). **Kai Sheng:** Formal analysis (supporting); methodology (supporting). **Yanping Li:** Formal analysis (supporting); funding acquisition (equal); methodology (supporting). **Peng Zhang:** Conceptualization (supporting); project administration (equal); resources (supporting); writing – review and editing (equal). **Xiugui Sheng:** Conceptualization (equal); funding acquisition (equal); project administration (equal); resources (equal); writing – review and editing (lead).

## CONFLICT OF INTEREST STATEMENT

The authors declare no competing interests.

## Supporting information


Figures S1–S4.

Table S1.

## Data Availability

Data available on request from the authors.

## References

[1] Sung H , Ferlay J , Siegel RL , et al. Global cancer statistics 2020: GLOBOCAN estimates of incidence and mortality worldwide for 36 cancers in 185 countries. CA Cancer J Clin. 2021;71:209‐249. 33538338 10.3322/caac.21660

[2] Siegel RL , Miller KD , Wagle NS , Jemal A . Cancer statistics, 2023. CA Cancer J Clin. 2023;73(1):17‐48. 36633525 10.3322/caac.21763

[3] Wang Y , Li BX , Li X . Identification and validation of angiogenesis‐related gene expression for predicting prognosis in patients with ovarian cancer. Front Oncol. 2022;11:783666. 35047401 10.3389/fonc.2021.783666 PMC8761815

[4] Wei X , Shi J , Lin Q , et al. Targeting ACLY attenuates tumor growth and acquired cisplatin resistance in ovarian cancer by inhibiting the PI3K–AKT pathway and activating the AMPK–ROS pathway. Front Oncol. 2021;11:642229. 33816292 10.3389/fonc.2021.642229 PMC8011496

[5] Wang S , Wang C , Liu O , Hu Y , Li X , Lin B . miRNA‐651‐3p regulates EMT in ovarian cancer cells by targeting ZNF703 and via the MEK/ERK pathway. Biochem Biophys Res Commun. 2022;619:76‐83. 35749939 10.1016/j.bbrc.2022.06.005

[6] Feng Q , Li X , Sun W , et al. Targeting G6PD reverses paclitaxel resistance in ovarian cancer by suppressing GSTP1. Biochem Pharmacol. 2020;178:114092. 32535103 10.1016/j.bcp.2020.114092

[7] Sheng H , Feng Q , Quan Q , Sheng X , Zhang P . Inhibition of STAT3 reverses Taxol‐resistance in ovarian cancer by down‐regulating G6PD expression in vitro. Biochem Biophys Res Commun. 2022;617(Pt 2):62‐68. 35689843 10.1016/j.bbrc.2022.05.091

[8] Sun CY , Nie J , Huang JP , Zheng GJ , Feng B . Targeting STAT3 inhibition to reverse cisplatin resistance. Biomed Pharmacother. 2019;117:109135. 31226634 10.1016/j.biopha.2019.109135

[9] Wang L , Liu X , Ren Y , et al. Cisplatin‐enriching cancer stem cells confer multidrug resistance in non‐small cell lung cancer via enhancing TRIB1/HDAC activity. Cell Death Dis. 2017;8(4):e2746. 28406482 10.1038/cddis.2016.409 PMC5477570

[10] Wu X , Zhao J , Ruan Y , Sun L , Xu C , Jiang H . Sialyltransferase ST3GAL1 promotes cell migration, invasion, and TGF‐beta1‐induced EMT and confers paclitaxel resistance in ovarian cancer. Cell Death Dis. 2018;9(11):1102. 30375371 10.1038/s41419-018-1101-0 PMC6207573

[11] Feng Y , Ma Z , Pan M , et al. WNT5A promotes the metastasis of esophageal squamous cell carcinoma by activating the HDAC7/SNAIL signaling pathway. Cell Death Dis. 2022;13(5):480. 35595735 10.1038/s41419-022-04901-x PMC9122958

[12] Guo K , Ma Z , Zhang Y , et al. HDAC7 promotes NSCLC proliferation and metastasis via stabilization by deubiquitinase USP10 and activation of β‐catenin‐FGF18 pathway. J Exp Clin Cancer Res. 2022;41(1):91. 35277183 10.1186/s13046-022-02266-9 PMC8915541

[13] Bai M , Cui M , Li M , et al. Discovery of a novel HDACi structure that inhibits the proliferation of ovarian cancer cells in vivo and in vitro. Int J Biol Sci. 2021;17(13):3493‐3507. 34512161 10.7150/ijbs.62339 PMC8416734

[14] Kwiecińska P , Wróbel A , Taubøll E , Gregoraszczuk EŁ . Valproic acid, but not levetiracetam, selectively decreases HDAC7 and HDAC2 expression in human ovarian cancer cells. Toxicol Lett. 2014;224(2):225‐232. 24200999 10.1016/j.toxlet.2013.10.035

[15] Caslini C , Hong S , Ban YJ , Chen XS , Ince TA . HDAC7 regulates histone 3 lysine 27 acetylation and transcriptional activity at super‐enhancer‐associated genes in breast cancer stem cells. Oncogene. 2019;38(39):6599‐6614. 31375747 10.1038/s41388-019-0897-0

[16] Feiteng Huang JS , Chen W , He X , et al. HDAC4 inhibition disrupts TET2 function in high‐risk MDS and AML. Aging. 2020;12:16759‐16774. 32726753 10.18632/aging.103605 PMC7521497

[17] Marroncelli N , Bianchi M , Bertin M , et al. HDAC4 regulates satellite cell proliferation and differentiation by targeting P21 and Sharp1 genes. Sci Rep. 2018;8(1):3448. 29472596 10.1038/s41598-018-21835-7 PMC5823886

[18] Cutano V , Di Giorgio E , Minisini M , Picco R , Dalla E , Brancolini C . HDAC7‐mediated control of tumour microenvironment maintains proliferative and stemness competence of human mammary epithelial cells. Mol Oncol. 2019;13(8):1651‐1668. 31081251 10.1002/1878-0261.12503 PMC6670296

[19] Zhang H , Wang Y , Dou J , et al. Acetylation of AGO2 promotes cancer progression by increasing oncogenic miR‐19b biogenesis. Oncogene. 2019;38(9):1410‐1431. 30305728 10.1038/s41388-018-0530-7 PMC6372475

[20] Zhang Y , Andrade R , Hanna AA , Pflum MKH . Evidence that HDAC7 acts as an epigenetic “reader” of AR acetylation through NCoR‐HDAC3 dissociation. Cell Chem Biol. 2022;29(7):1162‐1173.e1165. 35709754 10.1016/j.chembiol.2022.05.008 PMC9450512

[21] Wang R , Zhang H , Ding W , et al. miR‐143 promotes angiogenesis and osteoblast differentiation by targeting HDAC7. Cell Death Dis. 2020;11(3):179. 32152265 10.1038/s41419-020-2377-4 PMC7062786

[22] Li S , Wang B , Xu Y , Zhang J . Autotaxin is induced by TSA through HDAC3 and HDAC7 inhibition and antagonizes the TSA‐induced cell apoptosis. Mol Cancer. 2011;10(1):18. 21314984 10.1186/1476-4598-10-18 PMC3055229

[23] Peixoto P , Blomme A , Costanza B , et al. HDAC7 inhibition resets STAT3 tumorigenic activity in human glioblastoma independently of EGFR and PTEN: new opportunities for selected targeted therapies. Oncogene. 2016;35(34):4481‐4494. 26853466 10.1038/onc.2015.506

[24] Witt AE , Lee CW , Lee TI , et al. Identification of a cancer stem cell‐specific function for the histone deacetylases, HDAC1 and HDAC7, in breast and ovarian cancer. Oncogene. 2017;36(12):1707‐1720. 27694895 10.1038/onc.2016.337 PMC5364039

[25] Li Q‐G , Xiao T , Zhu W , et al. HDAC7 promotes the oncogenicity of nasopharyngeal carcinoma cells by miR‐4465‐EphA2 signaling axis. Cell Death Dis. 2020;11(5):322. 32376822 10.1038/s41419-020-2521-1 PMC7203158

[26] Ma Z‐Q , Feng Y‐T , Guo K , et al. Melatonin inhibits ESCC tumor growth by mitigating the HDAC7/β‐catenin/c‐Myc positive feedback loop and suppressing the USP10‐maintained HDAC7 protein stability. Mil Med Res. 2022;9(1):54. 36163081 10.1186/s40779-022-00412-0 PMC9513894

[27] Zhang Y , Ding P , Wang Y , et al. HDAC7/c‐Myc signaling pathway promotes the proliferation and metastasis of choroidal melanoma cells. Cell Death Dis. 2023;14(1):38. 36653340 10.1038/s41419-022-05522-0 PMC9849404

[28] Ha CH , Jhun BS , Kao H‐Y , Jin Z‐G . VEGF stimulates HDAC7 phosphorylation and cytoplasmic accumulation modulating matrix metalloproteinase expression and angiogenesis. Arterioscler Thromb Vasc Biol. 2008;28(10):1782‐1788. 18617643 10.1161/ATVBAHA.108.172528 PMC2746922

[29] Furumai R , Komatsu Y , Nishino N , Khochbin S , Yoshida M , Horinouchi S . Potent histone deacetylase inhibitors built from trichostatin a and cyclic tetrapeptide antibiotics including trapoxin. Proc Natl Acad Sci USA. 2001;98:87‐92. 11134513 10.1073/pnas.011405598 PMC14549

[30] Guardiola AR , Yao TP . Molecular cloning and characterization of a novel histone deacetylase HDAC10. J Biol Chem. 2002;277:3350‐3356. 11726666 10.1074/jbc.M109861200

[31] Mak JYW , Wu KC , Gupta PK , et al. HDAC7 inhibition by Phenacetyl and Phenylbenzoyl Hydroxamates. J Med Chem. 2021;64:2186‐2204. 33570940 10.1021/acs.jmedchem.0c01967

[32] Barneda‐Zahonero B , Collazo O , Azagra A , et al. The transcriptional repressor HDAC7 promotes apoptosis and c‐Myc downregulation in particular types of leukemia and lymphoma. Cell Death Dis. 2015;6(2):e1635. 25675295 10.1038/cddis.2014.594 PMC4669785

