# Supplementary material for: HDAC7 promotes ovarian cancer malignancy via AKT/mTOR signalling pathway
Source: J Cell Mol Med. 2024 Oct 21;28(20):e70120. doi: 10.1111/jcmm.70120 (PMC11491867; doi:10.1111/jcmm.70120)

Supplement 1. **The expression levels and survival analysis of HDAC7 in ovarian cancer cells**

1. B) TCGA data showed the expression levels (A) and survival analysis (B) of class IIa HDACs in ovarian cancer cells; (C) TCGA data showed the expression levels and survival analysis of HDAC7 in ovarian cancer cells; The data were expressed as mean ± standard deviation and analyzed by the t test (* P<0.05; **P<0.01; *** P<0.001).

Supplement 2. **HDAC7 promotes the activation of AKT via regulating mRNA expression of suppressors and activators in AKT signaling**

(A) A flag-pulldown experiment to detect the interaction between AKT and HDAC7; (B) The relative amount of differentially expressed mRNA both control and HDAC7 knockout groups in the heat map; (C) Volcano plot showed differentially expressed genes between two groups from control to HDAC7 knockout cells; (D) According to principal component analysis (PCA) of the relative abundance of mRNA expression both control and HDAC7 knockout groups. Significant differences across groups are established at the first principal component (PC1) values as shown in the box plots; (E) According to KEGG pathway, the changes of genes regulating AKT/mTOR signaling related pathways were analyzed by bulk RNA-seq analysis; (F) mRNA expression levels of KRAS, NRAS, PIK3C2B, PIK3CB, PIK3CD, GNG11, PPP2R2B and PTEN were assessed by qPCR; All experiments were repeated 3 times. The data were expressed as mean ± standard deviation and analyzed by the t test (* P<0.05; **P<0.01; *** P<0.001).

Supplement 3. **Reactivated AKT/mTOR signal pathway eliminates the effects of HDAC7 knockout in ovarian cancer cells**

(A) The levels of AKT/mTOR signal pathway, Flag and HDAC7 were measured by Western blot in HDAC7 knockout or HDAC7 knockout-AKT(T308D/S473D) and control groups. Cells with AKT(T308D/S473D) show rescue the level of and p-mTOR(S2448), compared to HDAC7 knockout groups; (B-D) The cell proliferation (B), Colony formation assay (C) and Transwell migration assay (D) of ES2 and OVCAR-3 cell lines which transfected HDAC7 knockout or HDAC7 knockout-AKT(T308D/S473D) lentiviral; The cell experiment was repeated 3 times. The data were expressed as mean ± standard deviation and analyzed by the t test (* P<0.05; **P<0.01; *** P<0.001).

Supplement 4. **Reactivated AKT/mTOR signal pathway eliminates the effects of HDAC7 knockout in vivo**

(A) NOD- Prkdc scid IL2rg tm1 /Bcgen mice were orthotopically injected with ES2 cells (1 × 10^6^ cells/mouse in 150 μl volume), which were infected with the indicated lentiviral vectors. Tumor volumes were measured by vernier caliper every three days until the end of the experiment; (B) The tumors were isolated and pictured (right) and tumour weight (left) was calculated in HDAC7 knockout or HDAC7 knockout-AKT(T308D/S473D) and control groups (n = 6 mice/group) for 30 days after injected; (C) The levels of AKT/mTOR signal pathway, Flag and HDAC7 were measured by Western blot in HDAC7 knockout or HDAC7 knockout-AKT(T308D/S473D) and control groups. Tumor tissue with AKT(T308D/S473D) show rescue the level of and p-mTOR(S2448), compared to HDAC7 knockout groups; The cell experiment was repeated 3 times. The data were expressed as mean ± standard deviation and analyzed by the t test (* P<0.05; **P<0.01; *** P<0.001).


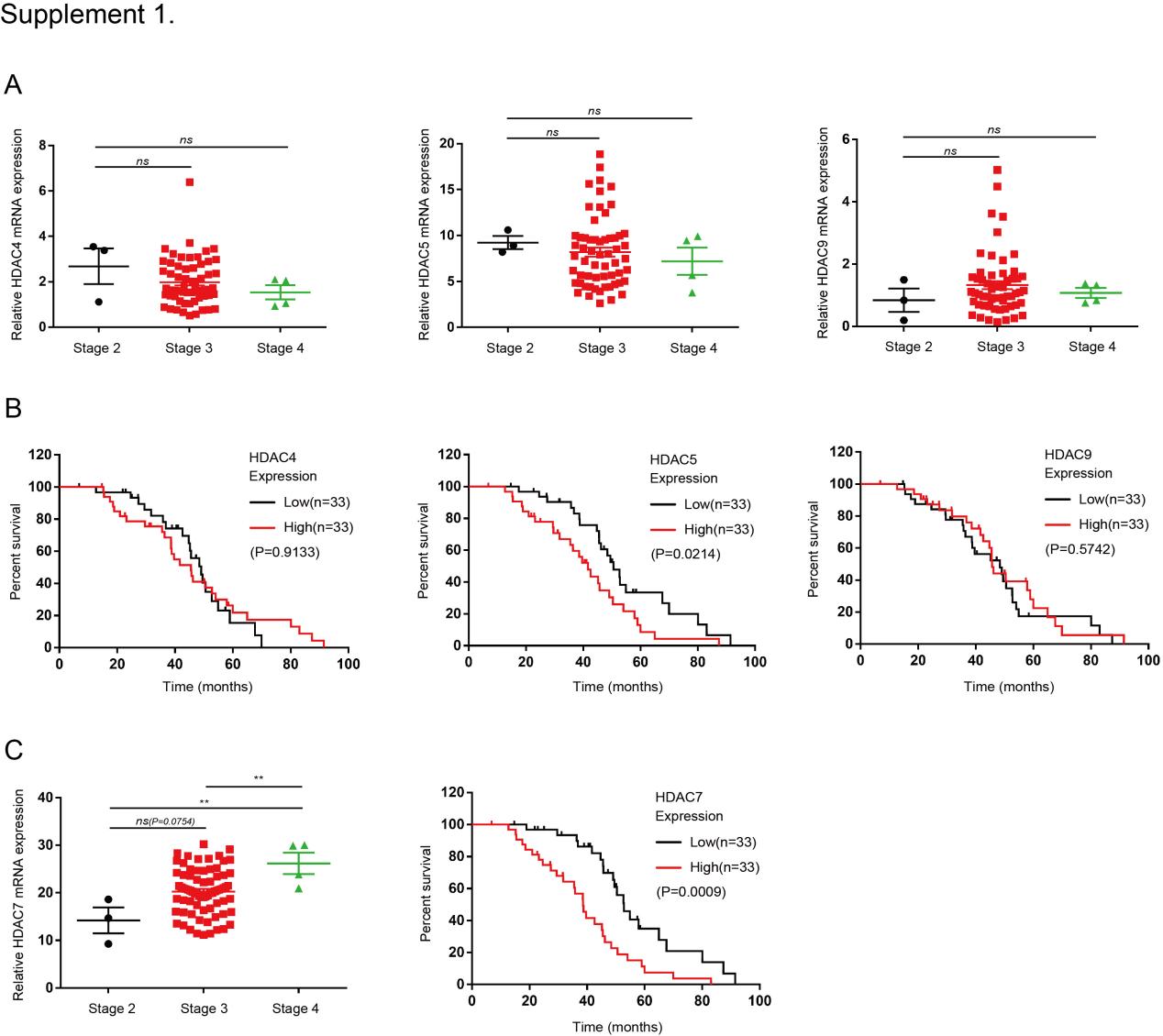


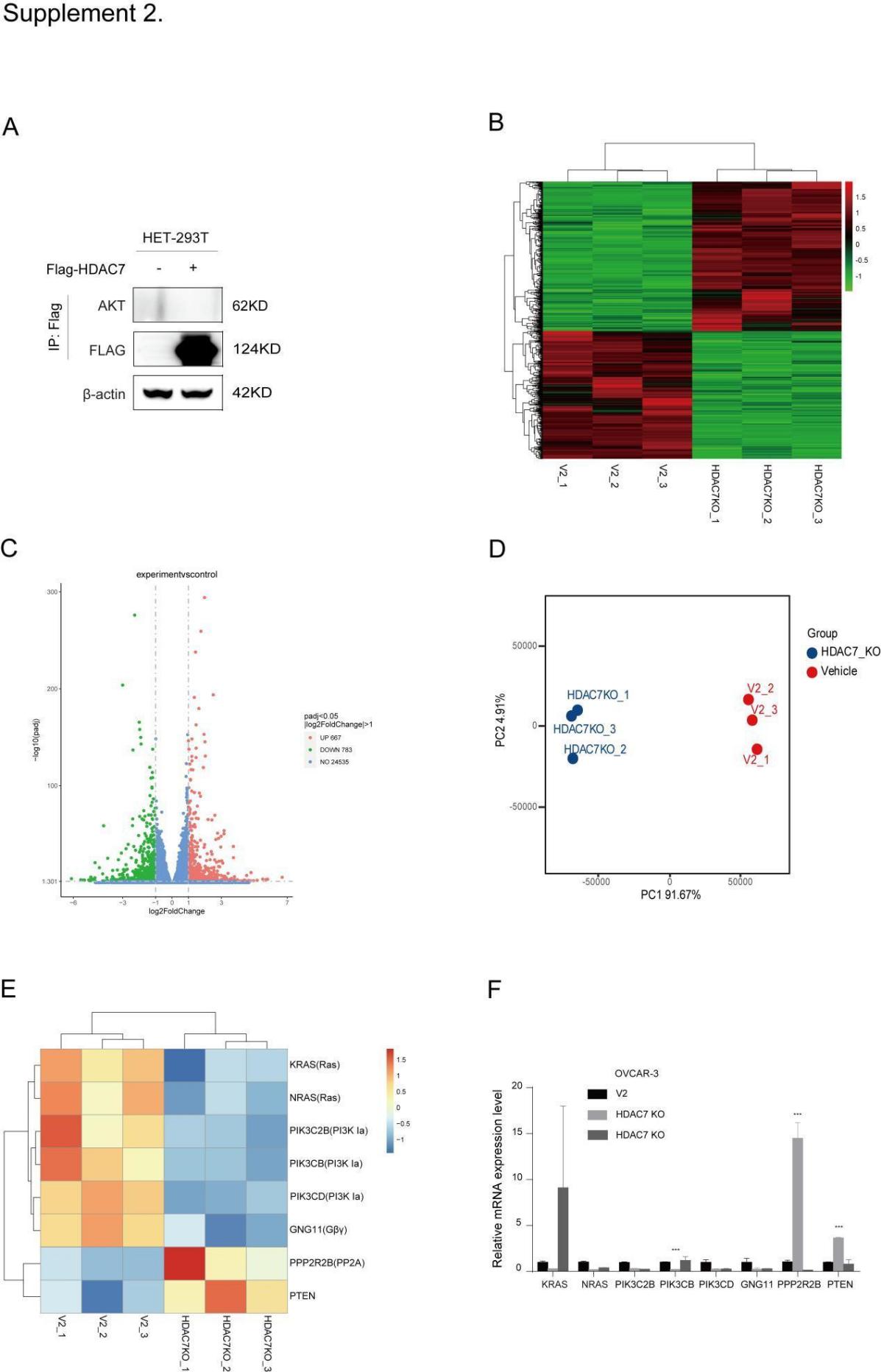

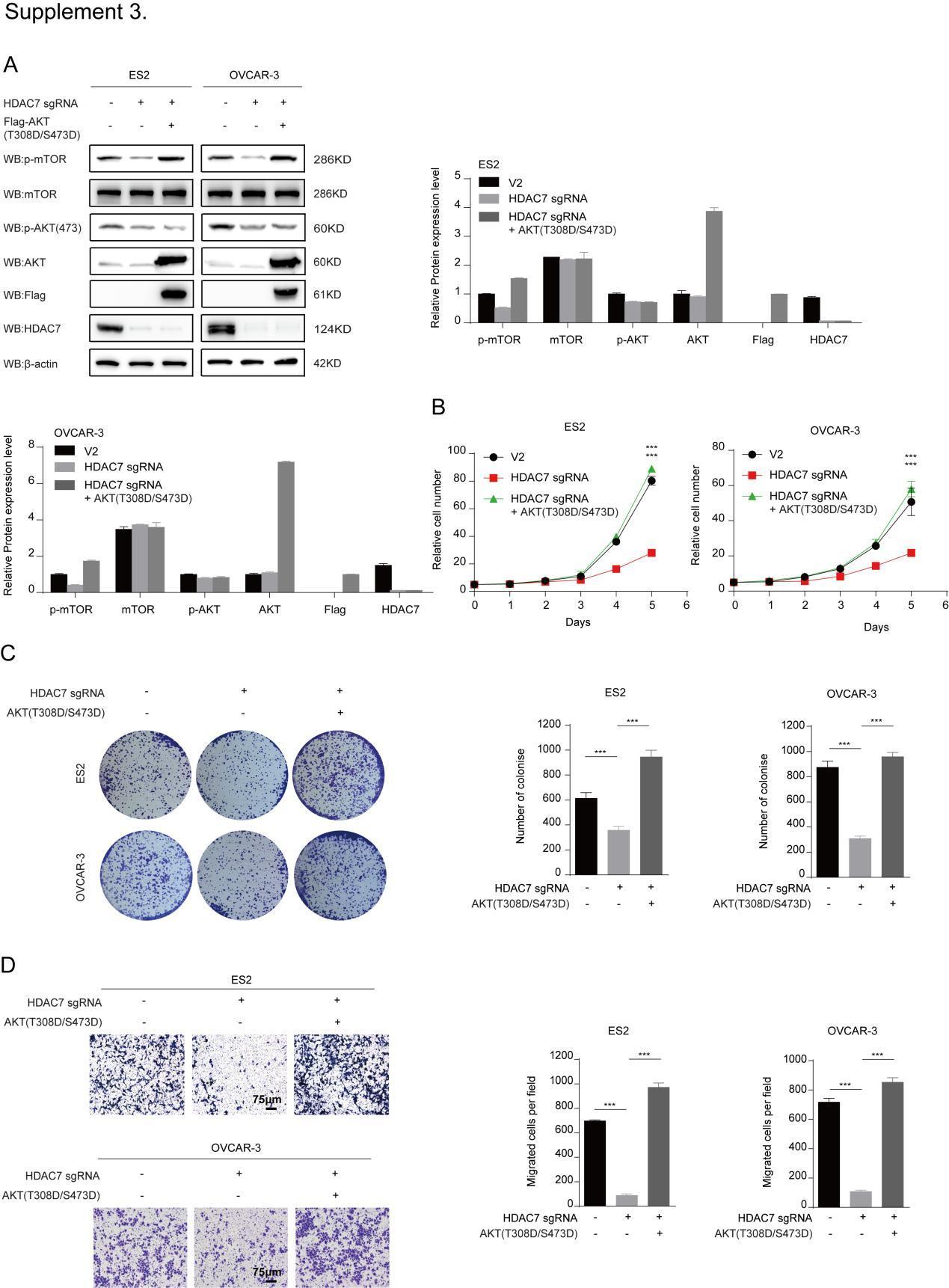

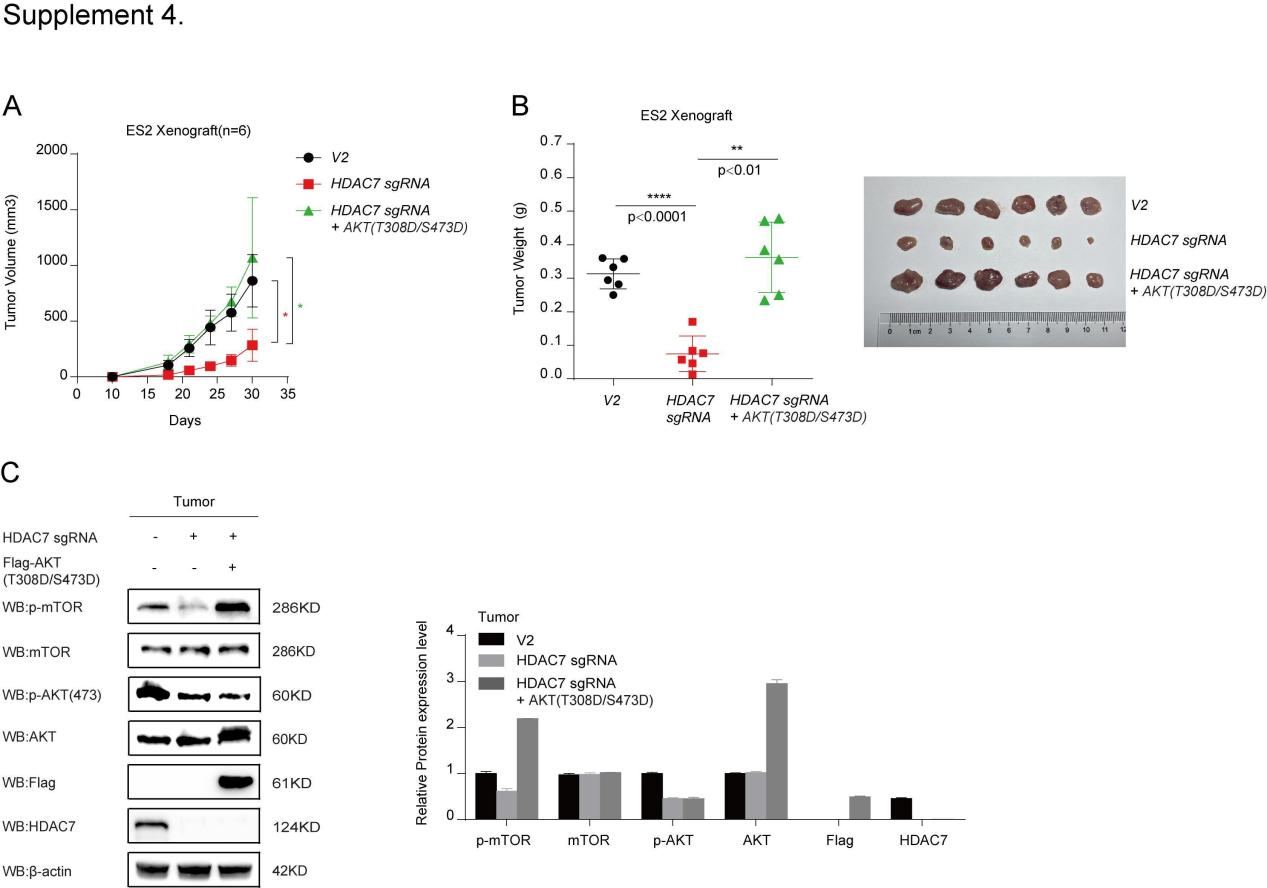

Supplement: Supplementary file 1 — Figures S1–S4. [file JCMM-28-e70120-s002.docx]
