# Supplementary material for: HDAC7 promotes ovarian cancer malignancy via AKT/mTOR signalling pathway
Source: J Cell Mol Med. 2024 Oct 21;28(20):e70120. doi: 10.1111/jcmm.70120 (PMC11491867; doi:10.1111/jcmm.70120)
Supplement: Supplementary file 2 — Table S1. [file JCMM-28-e70120-s001.docx]

Table S1.

Primers used in the study

| RT-qPCR primer | |
| --- | --- |
| Kras-Forward primer: | CAGTAGACACAAAACAGGCTCAG |
| Kras-Reverse primer: | TGTCGGATCTCCCTCACCAATG |
| Nras-Forward primer: | GAAACCTCAGCCAAGACCAGAC |
| Nras-Reverse primer: | GGCAATCCCATACAACCCTGAG |
| PIK3C2B-Forward primer: | CCTCCTGAAACGAGCTGTGTCT |
| PIK3C2B-Reverse primer: | CACAGTAAGGCTGCCAGCAGAT |
| PIK3CB-Forward primer: | GGTAATCGGAGGATAGGGCAGT |
| PIK3CB-Reverse primer: | CGGCAGTATGCTTCAAGGATGAC |
| PIK3CD-Forward primer: | TGCCAAACCACCTCCCATTCCT |
| PIK3CD-Reverse primer: | CATCTCGTTGCCGTGGAAAAGC |
| GNG11-Forward primer: | CTGCCCTTCACATCGAAGAT |
| GNG11-Reverse primer: | AAGGGGTTCTTGTCTTCTGGA |
| PPP2R2B-Forward primer: | ATGACTACCTCCGCAGCAAGCT |
| PPP2R2B-Reverse primer: | CATCACGCTTGGTGTTTCTGTCG |
| PTEN-Forward primer: | TGAGTTCCCTCAGCCGTTACCT |
| PTEN-Reverse primer: | GAGGTTTCCTCTGGTCCTGGTA |
| β-actin-Forward primer: | ACGTGGACATCCGCAAAG |
| β-actin-Reverse primer: | GACTCGTCATACTCCTGCTTG |
